# Advanced diffusion MRI provides evidence for altered axonal microstructure and gradual peritumoral infiltration in GBM in comparison to brain metastases

**DOI:** 10.1007/s00062-024-01416-0

**Published:** 2024-04-29

**Authors:** U. Würtemberger, A. Rau, M. Diebold, L. Becker, M. Hohenhaus, J. Beck, P. C. Reinacher, D. Erny, M. Reisert, H. Urbach, T. Demerath

**Affiliations:** 1 Department of Neuroradiology, Medical Center—University of Freiburg, Faculty of Medicine, University of Freiburg, 79106 Freiburg, Germany; 2 Department of Diagnostic and Interventional Radiology, Medical Center—University of Freiburg, Faculty of Medicine, University of Freiburg, 79106 Freiburg, Germany; 3 Institute of Neuropathology, Medical Center—University of Freiburg, Faculty of Medicine, University of Freiburg, 79106 Freiburg, Germany; 4 Department of Neurosurgery, Medical Center—University of Freiburg, Faculty of Medicine, University of Freiburg, 79106 Freiburg, Germany; 5 Fraunhofer Institute for Laser Technology, 52074 Aachen, Germany; 6 Department of Medical Physics, Medical Center—University of Freiburg, Faculty of Medicine, University of Freiburg, 79106 Freiburg, Germany; 7 Department of Stereotactic and Functional Neurosurgery, Medical Center—University of Freiburg, Faculty of Medicine, University of Freiburg, 79106 Freiburg, Germany; 8 Dept. of Neuroradiology, University Medical Center Freiburg, Breisacher Str. 64, 79106 Freiburg, Germany

**Keywords:** Glioblastoma, Metastasis, Diffusion Magnetic Resonance Imaging, Diffusion Tensor Imaging, Radiomics

## Abstract

**Purpose:**

In contrast to peritumoral edema in metastases, GBM is histopathologically characterized by infiltrating tumor cells within the T2 signal alterations. We hypothesized that depending on the distance from the outline of the contrast-enhancing tumor we might reveal imaging evidence of gradual peritumoral infiltration in GBM and predominantly vasogenic edema around metastases. We thus investigated the gradual change of advanced diffusion metrics with the peritumoral zone in metastases and GBM.

**Methods:**

In 30 patients with GBM and 28 with brain metastases, peritumoral T2 hyperintensity was segmented in 33% partitions based on the total volume beginning at the enhancing tumor margin and divided into inner, middle and outer zones. Diffusion Tensor Imaging (DTI)-derived fractional anisotropy and mean diffusivity as well as Diffusion Microstructure Imaging (DMI)-based parameters Dax-intra, Dax-extra, V‑CSF and V-intra were employed to assess group-wise differences between inner and outer zones as well as within-group gradients between the inner and outer zones.

**Results:**

In metastases, fractional anisotropy and Dax-extra were significantly reduced in the inner zone compared to the outer zone (FA *p* = 0.01; Dax-extra *p* = 0.03). In GBM, we noted a reduced Dax-extra and significantly lower intraaxonal volume fraction (Dax-extra *p* = 0.008, V‑intra *p* = 0.006) accompanied by elevated axial intraaxonal diffusivity in the inner zone (*p* = 0.035). Between-group comparison of the outer to the inner zones revealed significantly higher gradients in metastases over GBM for FA (*p* = 0.04) as well as the axial diffusivity in the intra- (*p* = 0.02) and extraaxonal compartment (*p* < 0.001).

**Conclusion:**

Our findings provide evidence of gradual alterations within the peritumoral zone of brain tumors. These are compatible with predominant (vasogenic) edema formation in metastases, whereas our findings in GBM are in line with an axonal destructive component in the immediate peritumoral area and evidence of tumor cell infiltration with accentuation in the tumor’s vicinity.

**Supplementary Information:**

The online version of this article (10.1007/s00062-024-01416-0) contains supplementary material, which is available to authorized users.

## Introduction

The noninvasive differentiation between glioblastoma (GBM) and brain metastases is challenging in conventional MRI. Both entities comprise comparable imaging features such as peritumoral T2-hyperintense signal alterations around a contrast-enhancing tumor. Whereas the primarily expansive growth pattern of metastases leads to peritumoral edema, GBM exhibits tumor infiltration into and even beyond peritumoral signal alterations [1, 2]. Due to the infiltrative growth pattern of GBM, recurrences frequently occur at the margins of the resection cavity [3]. Thus supratotal tumor resection beyond the contrast-enhanced tumor is increasingly considered [4]. Therefore, a more detailed investigation of the extent of tumor infiltration within the peritumoral zone is paramount.

Several studies have investigated the peritumoral T2 hyperintense zone in these entities seeking for imaging correlates of diffuse peritumoral infiltration in GBM: FET-PET can be used to visualize metabolically active tumor in GBM beyond the contrast-enhancing tumor [5], and widespread MR perfusion imaging provides evidence of possible tumor neoangiogenesis within the peritumoral T2 changes in GBM, which in comparison to metastatic tumors may present with increased peritumoral rCBV [6–8]. Comparative studies using diffusion-weighted MRI have revealed lower immediate peritumoral ADC-values in GBM [9, 10] and also, in general, a relatively increased mean diffusivity within peritumoral signal alterations in metastases [11]. In contrast to functional information on metabolism and vascularization, recent diffusion-weighted MRI techniques allow for the approximation of microstructural information in vivo from sequence parameters of millimeter-sized voxels in a mesoscopic approach [12]. The use of diffusion tensor imaging (DTI) and its parameters fractional anisotropy (FA) was frequently investigated [13], however, with varying results [14]. Several studies describe increased FA in peritumoral edema in GBM/high-grade gliomas compared to metastases [15] but there are also studies that find no significant differences in FA [16, 17].

Novel, biophysically motivated diffusion-weighted MRI techniques such as Diffusion Microstructure Imaging (DMI) are based on a multicompartment model, which disentangles the distribution of microstructural compartments per voxel [18]. The DMI model differentiates the three volume fractions V‑CSF (free water compartment), V‑intra (intraaxonal compartment) and V-extra (extraaxonal compartment, representing the extra-axonal cellular compartment and the extracellular matrix), as well as the corresponding diffusivities Dax-intra (axial intraaxonal diffusivity) and Dax-extra (axial extraaxonal diffusivity).

Unlike conventional MRI sequences, DMI allows for detecting previously masked information even in brain tissue with supposedly homogeneous signal representation in both inconspicuous and pathologically signal-altered structures. Compared to DTI, biophysically motivated multicompartment models like neurite orientation dispersion and density imaging (NODDI) and diffusion microstructure imaging (DMI) provide a more specific and interpretable approximation of the microstructure. For example, in T2 hyperintense lesions such as periventricular caps in NPH [19] or peritumoral edema around intracranial masses [11, 20] the proportionate free water content can be calculated. Moreover, structural alterations can be detected even in conventionally inconspicuous brain parenchymal structures in patients with mesial temporal sclerosis, subacute COVID-19 or Parkinson’s disease [21–23].

Regarding the peritumoral zone, DMI revealed significant differences in the free water content in GBM and metastases with elevated free water around metastases, matching histopathological considerations [20]. There were signs of increased, primarily vasogenic edema formation around metastases, while in GBM this was significantly less accentuated. Though the peritumoral T2 signal elevations were examined in whole, not taking into account the possibility of spatial gradual variations and heterogeneity. We hypothesize that gradual zone-based analysis using novel diffusion-weighted imaging metrics such as Dax-extra may detect differences between GBM and metastases that indicate an infiltrative tumor component in GBM.

Thus, we investigated the microstructure within the peritumoral zones of GBM and brain metastases using DTI and DMI in a mesoscopic approach.

## Materials and methods

### Patient and imaging characteristics

We enrolled patients with newly diagnosed GBM and brain metastases within 5 years (02/2018–12/2022). Histological analysis of surgically resected material was performed by standardized protocols of the local institute of neuropathology. Histopathological classification was done based on the WHO version 2016 in those patients examined prior to the release of the currently valid 2021 WHO CNS tumor classification. Patient demographics are summarized in Table 1. Patients with relevant small vessel disease (Fazekas > 1), concomitant vascular lesions (e.g., vascular malformations), or imaging features of neurodegenerative disorders (e.g., Alzheimer’s disease, frontotemporal lobar degeneration, cerebral amyloid angiopathy) were excluded. Similarly, previous tumor resections and brain biopsies, prior radiation therapy, or poor image quality led to study exclusion.

**Table 1:** Patient characteristics. Age is stated as mean and SD and perifocal T2-volume as median and interquartile ranges (IQR)

	GBM	Metastasis	*p*-value
*n*	30	28	**–**
Sex (m/f)	18/12	18/10	*p* = 0.74
Age (years) (SD)	64 (±10)	64 (±10)	*p* = 0.93
Perifocal T2-volume (ml) [IQR]	27.9 [16.9–43.7]	26.0 [4.1–39.7]	*p* = 0.29

All procedures received prior approval by the institutional review board and were in accordance with the 1964 Helsinki declaration and its later amendments. Informed written consent was obtained from all participants.

Imaging was conducted with 3 T MRI scanners (MAGNETOM Prisma and MAGNETOM Prisma FIT, Siemens Healthcare, Erlangen, Germany) using a 64-channel head and neck coil. T1-weighted (T1w) images were acquired 4–5 min after intravenous injection of 0.1 mmol/kg gadobutrol (ProHance®, Bracco Imaging, Milan, Italy) with three-dimensional (3D) magnetization-prepared 180° radio-frequency pulses and a rapid gradient-echo (MP-RAGE) sequence (repetition time 2500 ms; echo time 2.82 ms; flip angle 7°, TI = 1100 ms; GRAPPA factor = 2; 1.0 mm isotropic voxels; 160 contiguous sagittal slices). T2-weighted (T2w) isotropic 3D FLAIR images were acquired (repetition time 5000 ms; echo time 388 ms; flip angle variable; TI = 1800 ms; 1.0 mm isotropic voxels; 160 contiguous sagittal slices). Diffusion MRI sequences were acquired with the following parameters: axial orientation, 42 slices, voxel size 1.5 × 1.5 × 3 mm, repetition time 2800 ms, echo time 88 ms, bandwidth 1778 Hz, flip angle 90°, simultaneous multi-band acceleration factor 2, GRAPPA factor 2, 65 diffusion-encoding gradient directions, 15 non-diffusion weighted images, 2 × 58 images with b‑factors 1000 and 2000 s/mm^2^; acquisition time was 6:22 min.

### Image Postprocessing

Data processing was implemented in a local instance of the post-processing platform NORA (www.nora-imaging.org; last accessed on 24 October 2022). T1w image datasets were automatically segmented into white matter, gray matter, and cerebrospinal fluid (CSF) with SPM12 (Wellcome Centre for Human Neuroimaging, London, UK).

Preprocessing of diffusion MRI data included denoising [24], Gibbs-ringing artifacts-correction [25] and upsampling to isotropic resolution of 1.5 mm^3^ [18]. DTI measures were obtained from b = 0 and 1000 s/mm^2^ images using a publicly available open-source toolbox (https://www.uniklinik-freiburg.de/mr-en/research-groups/diffperf/fibertools.html) using the ordinary log-linear fitting, calculating the FA and MD. DMI metrics were obtained in a Bayesian approach [18, 20], extracting the intra-axonal (Dax intra) and extra-axonal diffusivities (Dax extra), and the volume fractions of free water (V-CSF) and the intra-axonal space (V-intra). The approach in [18] uses derived features of the signal rather than the raw signal itself. These signal features depend exclusively on microstructural tissue properties. The model is based on the assumption that in the intra-axonal compartment, water can diffuse along axons only, in the extra-axonal compartment it can diffuse both along, and radially to, the orientation of the axons, and in the free-fluid compartment, it can diffuse unrestrictedly in all directions. The diffusivity of the free-fluid compartment is fixed to 3 μm^2^/ms. This leads to a model, in which we report four parameters: The two compartmental volume fractions (V-intra, V‑CSF) and the two compartmental diffusivity parameters (Dax-intra, Dax-extra). The mathematical details of the model are described in [18].

**Fig. 1:**
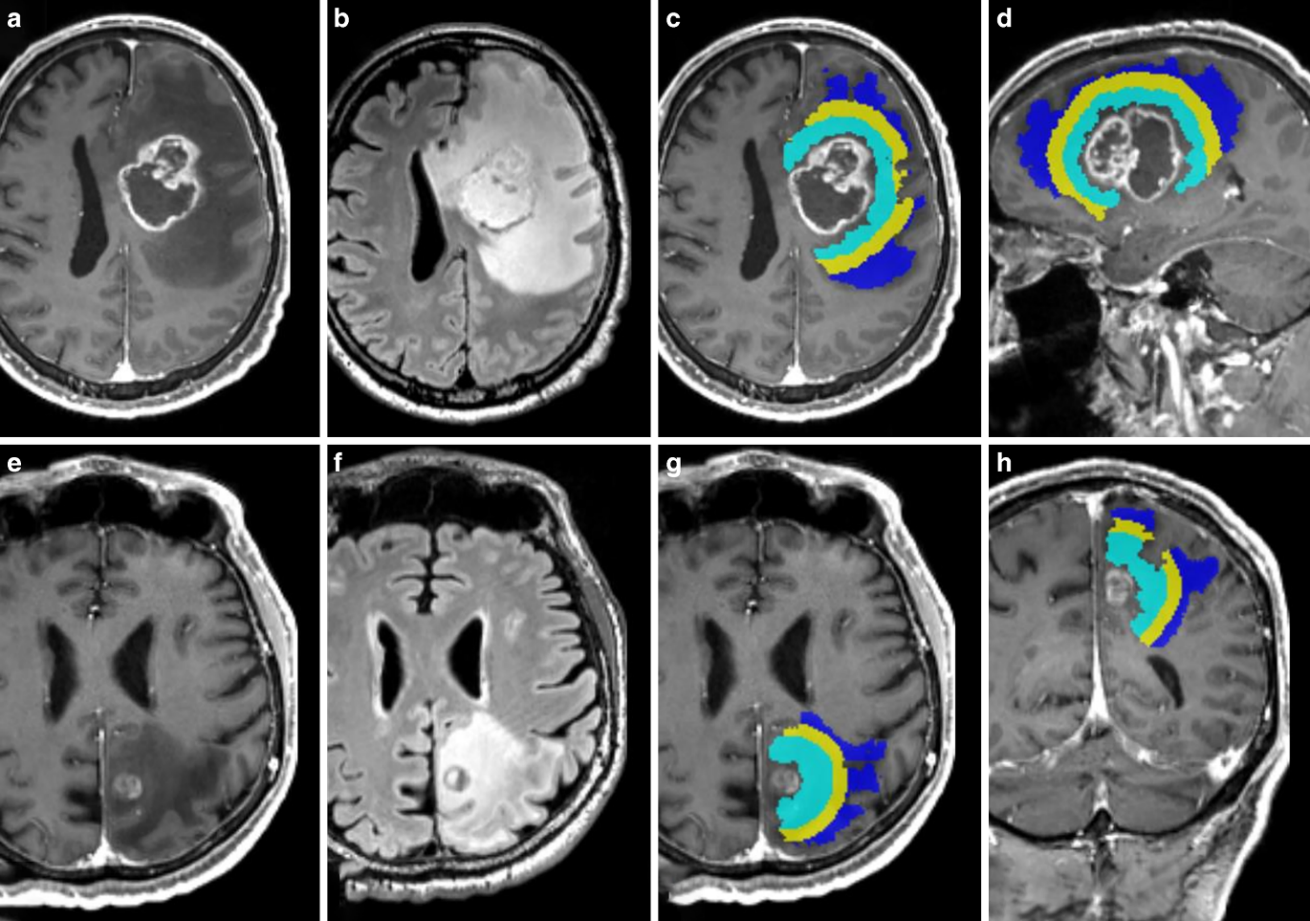
Axial T1 MPRAGE post-Gd (**a**,**c**,**d**,**e**,**g**,**h**) and FLAIR (**b**,**f**) images in a patient with a left frontal glioblastoma (upper row) and a left parietal metastasis (lower row) with corresponding color-coded subsegmentations of perilesional FLAIR-hyperintensity (**c**,**d**,**g**,**h**), parcellated in 33% zones of the total volume (innermost = IZ, turquoise; middle = MZ, yellow; and outermost zones = OZ in blue), sparing contrast-enhancing tumor components and cortex

Contrast-enhancing tumor (ceT) components were manually segmented by two neuroradiologists (with 5 and 7 years of experience in clinical neuroimaging) on 3D T1w post-Gd datasets in consensus. Peritumoral T2w hyperintense signal alterations were delineated on isotropic 3D T2w FLAIR images, co-registered to 3D T1w post-Gd datasets, carefully avoiding erroneous segmentation of contrast-enhancing tumor components. To account for potential partial volume effects, we carefully excluded contrast-enhancing outer tumor margins and adjacent gray matter (exemplary case presented in Fig. 1). In patients with more than one metastasis, only the largest lesion within the supratentorial white matter was selected for processing. Moreover, we carefully avoided an overlap of the metastasis’ peritumoral T2w signal alterations with that of another metastatic lesion.

From the outline of the ceT-mask, a perpendicular Euclidean expansion within the edema mask was done. Subsequently, the edema mask was parcellated in 33% partitions of the total volume beginning at the enhancing tumor margin and divided into inner, middle and outer zones, with the outermost zone (OZ) representing the boundary of the edema, and the innermost zone (IZ) adjacent to the ceT (Fig. 1). Mesoscopic diffusion metrics were read from the three subpartition zones of the edema and compared to each other.

### Statistical Analysis

The assumption of normal data distribution of the data was tested with Shapiro–Wilk test. Continuous variables are reported as mean and standard deviation or median and ranges/interquartile ranges (IQR) as appropriate. Comparisons of patient age and gradual changes of diffusion metrics in GBM vs. metastases was done using t‑test or the Mann–Whitney U test as appropriate. Sex was compared with Chi-square test between GBM and metastases groups. Within the respective group, diffusion metrics were compared between the inner and outer zone with paired samples t‑test or Wilcoxon rank test as appropriate. ANCOVA was conducted between gradual differences of diffusion metrics comparing metastases and GBM groups and controlling for the respective edema volume. An α‑level of 0.05 was considered statistically significant. Statistical analyses were performed using R statistics V. 4.0 (R Core Team 2020, Bell Laboratories, Holmdel, NJ, USA; https://www.R-project.org.) Boxplots were calculated using CRAN.R packages (https://CRAN.R-project.org/package=ggplot2, https://CRAN.R-project.org/package=ggstatsplot). Line diagrams were created with MS Excel 2016 (Microsoft Corporation, Redmont, USA).

## Results

### Study Population

A total of 58 patients consisting of *n* = 30 GBM (mean age 64 ± 10 years, 12 females) and *n* = 28 metastases (64 ± 10 years, 10 females) were included in this study. The primary tumors in the brain metastases group comprised non-small cell lung cancer (*n* = 15), small-cell lung cancer (*n* = 2), colorectal carcinoma (*n* = 1), ovarial carcinoma (*n* = 1), melanoma (*n* = 4), urothelial carcinoma (*n* = 2), breast cancer (*n* = 3). There was no significant difference in age (*p* = 0.93) nor sex (*p* = 0.74) nor edema volume (*p* = 0.29) between both groups.

### Characterization of the peritumoral edema in GBM and metastases

**Fig. 2:**
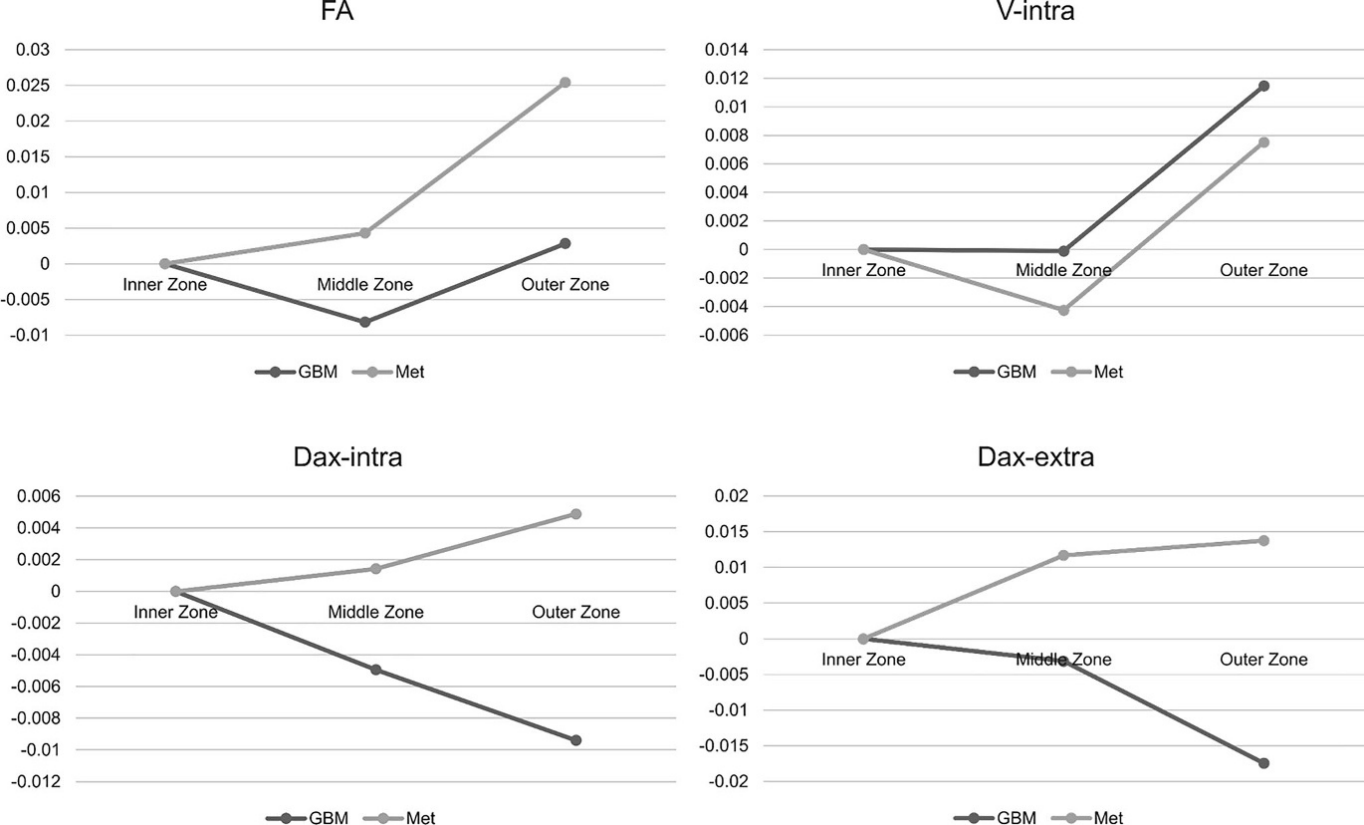
Gradual differences of inner, middle and outer peritumoral zones normalized to inner zone data, presented as differences inner—inner (IZ-IZ), middle—inner (MZ-IZ) and outer—inner (OZ-IZ) zones in GBM and metastases

Gradual differences between the intraindividual inner and outer zone were noted both in GBM and metastases (Fig. 2). In detail, DTI-derived FA was significantly lower in the IZ in metastases compared to the OZ (0.18 ± 0.04 vs. 0.19 ± 0.04; *p* = 0.01), while no significant effect in the GBM group was noted (*p* = 0.67). Further, Dax-extra was significantly lower in the IZ in both GBM (1.22 [1.21–1.25] vs. 1.23 [1.19–1.24]; *p* = 0.008) and metastases (1.24 [1.22–1.26] vs. 1.25 [1.24–1.27]; *p* = 0.03). Dax-intra was significantly higher in the IZ vs. OZ in GBM (2.29 [2.27–2.30] vs. 2.28 [2.27–2.29]; *p* = 0.035). V‑intra in GBM differed significantly between the inner and outer zone with a lower intra-axonal volume fraction in the IZ (0.08 [0.07–0.11] vs. 0.09 [0.07–0.12]; *p* = 0.006). No significant gradual alterations between the inner and outer zone were noted for the other diffusion metrics including the free-water sensitive V‑CSF and MD (all *p* > 0.05). However, in GBM between IZ and OZ a trend towards a peripheral decrease in MD (1.11 ± 0.15 vs. 1.08 ± 0.17; *p* = 0.152) and V-CSF (0.48 ± 0.12 vs. 0.47 ± 0.13; *p* = 0.186) was present.

### Gradual differences in the peritumoral zone in GBM vs. metastases

**Fig. 3:**
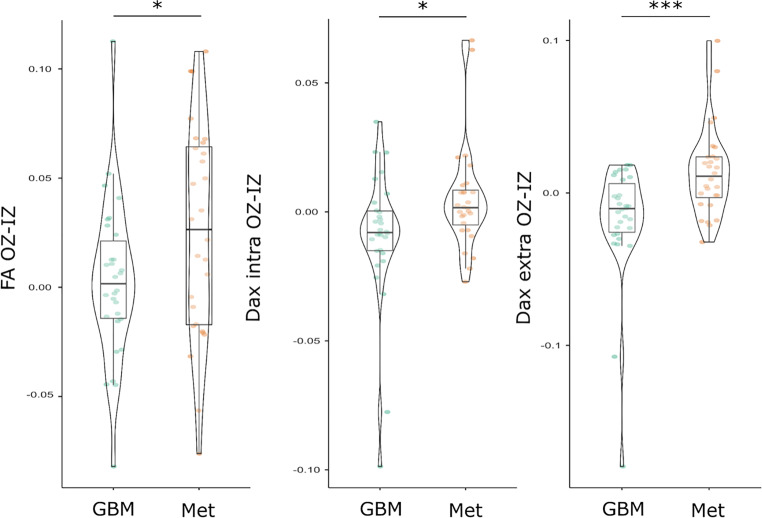
Gradual differences in fractional anisotropy (FA), axial intraxonal diffusivity (Dax-intra), and axial extraaxonal diffusivity (Dax-extra), between the outer (OZ) and inner (IZ) peritumoral T2-hyperintense zones. * *p* < 0.05; *** *p* < 0.001

The difference of FA between the outer and inner zone (OZ-IZ) was significant in GBM and metastases (0.003 ± 0.04 vs. 0.03 ± 0.05; *p* = 0.049). Further significant differences were noted for Dax-intra (OZ-IZ) (−0.008 [−0.015–0.0004] vs. 0.002 [−0.005–0.008]; *p* = 0.02) and Dax-extra (OZ-IZ) (−0.01 [−0.026–0.006] vs. 0.01 [−0.003–0.024]; *p* < 0.001). These results are illustrated in Fig. 3. After controlling for edema volume, differences between GBM and metastases remained significant for FA (*p* = 0.04), Dax-intra (*p* = 0.02), and Dax-extra (*p* < 0.001).

## Discussion

Advanced diffusion-weighted imaging revealed gradual microstructural differences within the peritumoral edema of GBM and metastases, which may be attributed to differing histopathological characteristics of these zones.

Although there was no significant difference in free water between the inner and outer peritumoral zones, there is evidence that subtle effects of free water decreasing peripherally is present at least in GBM, where we found a trend towards declining MD and V-CSF from the IZ to the OZ but without reaching significance. Beyond this, FA significantly increased outward in metastases and, on the other hand, in both entities V‑intra increased outward. According to the three-compartment theory, this is indirectly linked to a decrease in the other components [18], even if a level of significance in those was not reached. The larger FA gradient in metastases also distinguished them from GBM, even after controlling for edema size. Previous studies focusing on peritumoral diffusion imaging corroborate our findings, as Latysheva et al. [26] did not observe a significant difference between median FA or MD values between inner and outer peritumoral zones in GBM. Wang et al. [17] did not observe a significant between-group difference regarding GBM and metastases groups considering inner or outer peritumoral ADC (Apparent Diffusion Coefficient), but also not for FA. Thus, the available data hint at the limited use of MD and V-CSF to further stratify the peritumoral infiltration zone in these entities, even if metastases and GBM differ in terms of mean global peritumoral water content, as previously demonstrated using V‑CSF [20]. This primarily corresponds to the different edema characteristics with a predominantly vasogenic edema for metastases and an additional infiltrative edema component for GBM.

The discrepancy of FA between inner and outer zones in metastases suggests an additional tumor related effect in GBM beyond the vasogenic edema component. This may be related to peripheral tumor infiltration on the one hand, and indirect effects alternating the axonal microstructure on the other, ultimately “preventing” peripheral normalization of FA levels. The significantly decreased V‑intra in the inner zone in GBM is also indicative of axonal damage. This is plausible as GBM commonly present with extensive necrosis affecting both gray and white matter [27] leading to secondary wallerian degeneration of the axons involved. Metastases commonly exhibit expansional growth, even though tumor infiltration within the immediate inner peritumoral areas has been reported [28]. This is supported by the finding that Dax-intra was relatively increased in GBM in the inner vs. the outer zone: It is known that structural harm of the axon may not only lead to axonal loss but also an increase of the axons’ inner diameter, which has been indicated by histopathological studies in multiple sclerosis [29], in acute ischemic stroke [30], and may also occur in chronic traumatic brain injury [31].

In contrast, no significant difference was detectable in metastases, which suits well with a pure vasogenic edema component with no or minimal impairment of the axons passing through.

The most interesting finding ultimately emerges in the analysis of the Dax-extra: On the one hand, Dax-extra was reduced in both groups in the inner zone, but on the other hand, a divergent effect emerged (similar to that related to Dax-intra) when considering the difference of the inner vs. outer zones. This divergence might fit to the fact that in the inner zone of metastases, a predominant space-occupying/compressing effect is present, whereas in primarily infiltrating glioblastomas a more pronounced Dax-extra reduction in the peritumoral inner zone leads to a negative gradient (Out-In). This may be caused by a pronounced mass-effect. However, as GBM usually represent diffusely infiltrating lesions, these findings may rather be explained by infiltrating elements along the axon/axonal sheath [27], limiting axial diffusion along those. This finding is supported by previous studies indicating a gradually decreasing cellularity from in- to outside in GBMs in contrast to metastases [32]. Besides the perivascular and leptomeningeal spaces, the brain parenchyma and white matter tracts represent main routes of invasion in GBM [33] with molecular mechanisms so far being poorly understood. Since structural MRI biomarkers of axonal tumor spread are largely lacking, Dax-extra might be an interesting candidate warranting further investigations.

Compared to PWI and PET, diffusion imaging represents a less invasive approach, as it eliminates the need for contrast agent administration and does not involve radiation exposure. Additionally, advanced diffusion-weighted imaging is part of standard cerebral MRI protocols in primary care centers, and the sequence, as outlined in the methods section and Supplementary Table 1, does not substantially extend the overall acquisition time. Furthermore, for tumors located in eloquent areas, presurgical visualization of fiber tracts is crucial to avoid damaging critical structures such as the optic tract or corticospinal tract (CST) during resection. Thus, both microstructural diffusion MRI and tractography may be acquired within one sequence. From a therapeutic point of view, it would be conceivable to adapt surgical and/or postoperative radiotherapy with the aim of treating suspicious areas more aggressively. From a diagnostic point of view, an adjustment of the follow-up intervals would also be conceivable, depending on the quantitative findings.

A novel approach of our study is also that we captured and segmented the entirety of the peritumoral edema, while for example the DWI-based work of Lemercier et al. [9] manually selected three ROIs and the perfusion study by Aparici-Robles et al. [6] defined three adjacent zones of 3 mm diameter each around the contrast-enhancing tumor and thus did not account for the outer parts, in particular in extensive peritumoral signal alterations.

The study’s limitations certainly include undersampling, necessitating confirmation of results in a larger patient population. The drawn conclusions primarily rely on theoretical considerations, emphasizing the need for histopathologic correlation. However, obtaining tissue samples from peritumoral areas poses ethical challenges and additional procedural risks. The observed effects on microstructural diffusion imaging metrics encompass various peritumoral compartments with tumors of diverse genetic signatures and metastases exhibiting varying histopathology. Future investigations should address and potentially correct these heterogeneities. Notably, the measured values lack a connection to underlying white matter connectivity. The study acknowledges the possibility of distant axonal damage influencing distant white matter microstructure, highlighting the need for dedicated studies to explore these relationships. Additionally, the impact of connectivity on the presented outcomes, whether positive or negative, remains unclear and warrants further investigation.

In summary, our results indicate microstructural discrepancies in peritumoral brain tissue between GBM and metastases, with evidence of altered intraaxonal diffusivity and tumor cell infiltrates in GBM.

## Conclusions

Diffusion microstructural imaging of peritumoral white matter in GBM and metastases indicates axonal damage in the immediate peritumoral zone and microscopic tissue infiltration by tumor cells in GBM. This enables stratification of the peritumoral zone in these entities. These findings may have both diagnostic and therapeutic implications in the future, as more precise stratification of the peritumoral zone appears feasible. However, histopathological validation is warranted.

### Supplementary Information

**Supplementary Table 1**: Tumor-dedicated MRI protocol (3-Tesla MAGNETOM Prisma/Prisma fit, Siemens Healthcare, Erlangen, Germany).

**Supplementary Table 2**: DTI/DMI metrics obtained from inner, middle and outer peritumoral zones in GBM and metastases

## Data Availability

The anonymized data presented in the study are available on reasonable request from the corresponding author

## Abbreviations

ADC: Apparent Diffusion Coefficient
Dax-intra: axial intraaxonal diffusivity
Dax-extra: axial extraaxonal diffusivity
DMI: Diffusion Microstructure Imaging
FA: Fractional Anisotropy
IZ: Inner peritumoral zone
MD: Mean Diffusivity
MZ: Middle peritumoral zone
OZ: Outer peritumoral zone
rCBV: relative cerebral blood volume
V‑intra: intraaxonal volume fraction
V‑CSF: CSF/free water volume fraction
