# Supplementary material for: Advanced diffusion MRI provides evidence for altered axonal microstructure and gradual peritumoral infiltration in GBM in comparison to brain metastases
Source: Clin Neuroradiol. 2024 Apr 29;34(3):703–11. doi: 10.1007/s00062-024-01416-0 (PMC11339137; doi:10.1007/s00062-024-01416-0)
Supplement: Supplementary file 1 — Supplementary Table 1: Tumor-dedicated MRI protocol (3-Tesla MAGNETOM Prisma/Prisma fit, Siemens Healthcare, Erlangen, Germany). [file 62_2024_1416_MOESM1_ESM.docx]

**Supplementary Table 1**: Tumor-dedicated MRI protocol (3-Tesla MAGNETOM Prisma/Prisma fit, Siemens Healthcare, Erlangen, Germany).

| **MRI sequence** | **No. of slices/ thickness (mm)** | **Voxel size (mm^3^)** | **TI/TR/TE/α**  **(ms/ms/ms/°)** | **acquisition time (min:sec)** |
| --- | --- | --- | --- | --- |
| **Ax T2 DWI-SE EPI** | 23/5 | 0.6 x 0.6 x 5 | 3400/85 | 0:46 |
| **sag 3D FLAIR-SPACE** | 192/1 | 1x1x1 | 1800/5000/388/var | 6:52 |
| **ax 2D T2-TSE** | 42/3 | 0.4x0.4x3 | 5040/102/150 | 4:34 |
| **ax 2D T1 TIRM** | 23/5 | 0.7x0.7x3 | 900/2000/9/150 | 2:54 |
| **DTI/DMI** | 42/3 | 1.5 x 1.5 x 3 | -/2800/88/90 | 6:22 |
| **sag 3D MPRAGE (post-Gd)** | 160/ 1 | 1x1x1 | 1100/2500/2.82/7 | 3:58 |

EPI = Echo Planar Imaging, DMI = Diffusion-Microstructure Imaging, DTI = Diffusion Tensor Imaging, FLAIR SPACE = Fluid-Attenuated Inversion Recovery - sampling perfection with application-optimized contrasts by using flip angle evolution, MPRAGE = Magnetization Prepared Rapid Gradient Echo, TSE =Turbo Spin Echo, SE Spin Echo, TI = inversion time, TR = repetition time, TE = echo time, α = flip angle
