# Supplementary material for: Advanced diffusion MRI provides evidence for altered axonal microstructure and gradual peritumoral infiltration in GBM in comparison to brain metastases
Source: Clin Neuroradiol. 2024 Apr 29;34(3):703–11. doi: 10.1007/s00062-024-01416-0 (PMC11339137; doi:10.1007/s00062-024-01416-0)
Supplement: Supplementary file 2 — Supplementary Table 2: DTI/DMI metrics obtained from inner, middle and outer peritumoral zones in GBM and metastases [file 62_2024_1416_MOESM2_ESM.docx]

**Supplementary Table 2**: DTI/MI metrics obtained from inner, middle and outer peritumoral zones in GBM and metastases

|  |  | **FA IZ** | **FA MZ** | **FA OZ** | **MD IZ** | **MD MZ** | **MD OZ** | **Dax- intra IZ** | **Dax- intra MZ** | **Dax- intra OZ** | **Dax- extra IZ** | **Dax- extra MZ** | **Dax-extra OZ** | **V-CSF IZ** | **V-CSF MZ** | **V-CSF OZ** | **V-intra IZ** | **V-intra MZ** | **V-intra OZ** |
| --- | --- | --- | --- | --- | --- | --- | --- | --- | --- | --- | --- | --- | --- | --- | --- | --- | --- | --- | --- |
| Mean | GBM | 0.206 | 0.198 | 0.209 | 1.110 | 1.120 | 1.080 | 2.290 | 2.280 | 2.280 | 1.230 | 1.230 | 1.210 | 0.484 | 0.495 | 0.467 | 0.104 | 0.103 | 0.115 |
|  | Met | 0.176 | 0.180 | 0.201 | 1.140 | 1.150 | 1.110 | 2.280 | 2.280 | 2.280 | 1.240 | 1.250 | 1.250 | 0.526 | 0.544 | 0.517 | 0.088 | 0.084 | 0.095 |
| Median | GBM | 0.189 | 0.178 | 0.193 | 1.120 | 1.140 | 1.110 | 2.290 | 2.280 | 2.280 | 1.230 | 1.230 | 1.220 | 0.499 | 0.500 | 0.495 | 0.084 | 0.078 | 0.091 |
|  | Met | 0.177 | 0.178 | 0.192 | 1.140 | 1.160 | 1.110 | 2.270 | 2.280 | 2.280 | 1.240 | 1.260 | 1.250 | 0.522 | 0.544 | 0.531 | 0.082 | 0.081 | 0.095 |
| SD | GBM | 0.108 | 0.107 | 0.105 | 0.155 | 0.158 | 0.165 | 0.031 | 0.027 | 0.040 | 0.033 | 0.036 | 0.048 | 0.123 | 0.126 | 0.130 | 0.056 | 0.059 | 0.073 |
|  | Met | 0.039 | 0.027 | 0.040 | 0.130 | 0.104 | 0.101 | 0.018 | 0.013 | 0.017 | 0.037 | 0.033 | 0.033 | 0.093 | 0.076 | 0.076 | 0.033 | 0.029 | 0.030 |
| IQR | GBM | 0.059 | 0.067 | 0.056 | 0.208 | 0.225 | 0.179 | 0.023 | 0.020 | 0.021 | 0.046 | 0.050 | 0.058 | 0.177 | 0.172 | 0.138 | 0.042 | 0.034 | 0.046 |
|  | Met | 0.057 | 0.046 | 0.063 | 0.148 | 0.088 | 0.127 | 0.026 | 0.016 | 0.025 | 0.043 | 0.038 | 0.028 | 0.115 | 0.072 | 0.091 | 0.037 | 0.019 | 0.035 |

Dax-intra = axial intraaxonal diffusivity, Dax-extra = axial extraaxonal diffusivity, FA = fractional anisotropy, IZ = Inner peritumoral zone, MD = mean diffusivity, MZ = Middle peritumoral zone; OZ = Outer peritumoral zone;  V-intra = intraaxonal volume fraction, V-CSF = CSF/free water volume fraction
